# Ramadan intermittent fasting is associated with ameliorated inflammatory markers and improved plasma sphingolipids/ceramides in subjects with obesity: lipidomics analysis

**DOI:** 10.1038/s41598-023-43862-9

**Published:** 2023-10-13

**Authors:** Mohamed Ibrahim Madkour, Md Torikul Islam, Trevor S. Tippetts, Kamrul H. Chowdhury, Lisa A. Lesniewski, Scott A. Summers, Falak Zeb, Dana N. Abdelrahim, Refat AlKurd, Husam M. Khraiwesh, Katia H. AbuShihab, Asma AlBakri, Khaled Obaideen, MoezAlIslam E. Faris

**Affiliations:** 1 Department of Medical Laboratory Sciences, College of Health Sciences, University of Sharjah, Sharjah, UAE; 2 Department of Nutrition and Integrative Physiology, University of Utah, Salt Lake City, UT USA; 3 Research Institute of Medical and Health Sciences (RIMHS), University of Sharjah, Sharjah, UAE; 4 Department of Nutrition, Faculty of Pharmacy and Medical Sciences, University of Petra, Amman, Jordan; 5 Department of Nutrition and Food Processing, Faculty of Agricultural Technology, Al-Balqa Applied University, Salt, Jordan; 6 Department of Nutrition and Food Technology, School of Agriculture, The University of Jordan, Amman, Jordan; 7 Sustainable Energy and Power Systems Research Centre, RISE, University of Sharjah, Sharjah, UAE; 8 Department of Clinical Nutrition and Dietetics, College of Health Sciences, University of Sharjah, Sharjah, UAE

**Keywords:** Translational research, Sphingolipids

## Abstract

Intermittent fasting (IF) is associated with enormous metabolic alterations that underpin its diverse health effects. Changes in lipid metabolism, particularly ceramides, and other sphingolipids, are among the most notable of these alterations. This study investigated the lipidomic alterations associated with 29–30 days of Ramadan diurnal intermittent fasting (RIF) in metabolically healthy overweight and obese subjects. A prospective cohort of 57 overweight and obese adults (70% males, 38.4 ± 11.2 years), with an age range of 18–58 years was observed prior to and at the conclusion of Ramadan. At both time points, anthropometric, biochemical (lipid profile, glycemic, and inflammatory markers), and dietary intake measurements were taken. Using liquid chromatography-mass spectrometry, a lipidomic analysis of ceramides and other sphingolipids was conducted. Using paired sample t-tests, pre- and post-Ramadan anthropometric, biochemical, and dietary values were compared. RIF was associated with improved levels of lipid profile compartments and inflammatory markers. In addition, RIF was associated with a decrease in plasma sphingosine and sphinganine, which was accompanied by a decrease in sphingosine 1-phosphate and sphinganine 1-phosphate. In addition, RIF was associated with decreased C17, C22, and C24 sphingomyelin, but not C14, C16, C18, C20, and C24:1 sphingomyelin, as well as C20, C22, C24, and C24:1 dihydrosphingomyelin, but not C16 and C18 dihydrosphingomyelin. This study demonstrates that RIF is associated with improvements in plasma sphingosine, sphinganine sphingomyelin, and dihydrosphingomyelin lipid species, as well as improved lipid profile and inflammatory markers, which may confer short-term protection against cardiometabolic problems in patients with overweight/obesity.

## Introduction

Lipids are integral components of the human body with versatile and vital physiological functions such as energy storage and cellular signaling and represent integral parts of cellular membranes and lipid particles such as exosomes and lipoproteins^1^. Lipidomics analysis is a novel advanced analytical technique that enables studying the lipid metabolism in human health and disease conditions^2^. Sphingosine and a fatty acid make up the family of lipid molecules known as ceramides (Cer), which are carried throughout the bloodstream by lipoproteins, especially low-density lipoproteins^3^. They serve as both prospective biomarkers of cardiometabolic disorders as well as pharmaceutical targets^4^. They are not only structural lipids but also multifunctional and bioactive molecules with significant involvement in many crucial cellular pathways, such as inflammatory processes^3,4^, diabetes, insulin resistance, fatty liver disease^5^, hypertension, and cerebro- and cardiovascular diseases (CVD)^4^. These obesity-related illnesses are caused in part by the abnormal buildup of toxic lipid metabolites in tissues that are not designed to store lipids (e.g., the liver, vasculature, heart, and pancreatic β-cells)^6^. Particular ceramides have been associated with the risk of CVD, with positively associated species: phosphatidylcholine (PC) 16, PC 16:0/16:0; C18 Cer, Cer d(18:1, 18:0); C24:1 Cer, Cer d(18:1, 24:1); C16 Cer, Cer d(18:1, 16:0) and negatively associated species: C24, Per d(18:1, 24:0); PC22:5, PC 16:0/22:5; PC 36:6^7^.

Fasting, the abstinence from drinking or eating for a specific time, is one of the most commonly observed dietary modifications by humans since ancient times, driven by several health behaviors, religious, and spiritual motives^8^. Lately, growing attention has been directed toward intermittent fasting (IF) (a dietary pattern that involves periods with very little or no food) as a suggested safe, disease-preventing, health-improving, and cost-effective regimen. Extensively detailed reviews recently unraveled that IF has a wide spectrum of positive effects on aging and neurodegenerative diseases, cardiometabolic dysfunction, and vascular problems. Therefore, IF continues to gain attention as a preventative and therapeutic intervention to counteract such chronic diseases^9^. Considering the drastic shift in lifestyle and dietary behaviors during the practice of IF, a wide range of physiological implications with distinctive metabolic switches are induced^9,10^. Several models of IF have been examined for their assumed protective effect in alleviating cardiometabolic risk factors^11^, including time-restricted eating/feeding^12^, periodic fasting, fasting-mimicking diet^13^, and alternate-day fasting^14^, with consistent positive effects reported.

Among the types of IF, Ramadan intermittent fasting (RIF) is one of the most commonly observed religious forms of IF^10^. It is an obligatory practice imposed during the ninth month of the lunar calendar on adult Muslims. Intermittent fasting during Ramadan is observed by more than 1.5 billion Muslims throughout the world and is currently considered one of the most extensively studied forms of religious fasting^15^. Owing to its unique pattern of dry and diurnal fasting for 29–30 consecutive days from predawn to sunset^16^, the model of RIF has shown a plethora of beneficial anthropometric, metabolic, and inflammatory effects. These include improving body mass, dimensions, and composition^17,18^, alleviating metabolic syndrome components^18^, ameliorating inflammatory and oxidative stress markers^19,20^, normalizing glucose homeostasis^21^ and liver function markers^22^, and lessing cardiometabolic risk factors^22^ in healthy subjects.

During Ramadan fasting month, liberal eating is allowed during the night hours, without restriction on any of the allowed foods, with an eating window of 7–12 h (corresponding to 12–17 h of daily fasting based on the solar season that crosses with the lunar month of Ramadan). With the significant lifestyle and dietary changes associated with Ramadan month, including food groups and macronutrients^23^, sleep quality and duration^24^, and physical activity^25^ in comparison with the non-fasting days; it is hypothesized that RIF will be accompanied by distinctive lipidomic fingerprints that distinguish it from non-fasting days. Recent studies using proteomic analysis demonstrated that the dawn-to-sunset model of RIF for thirty consecutive days results in distinctive fingerprint modifications in anticancer proteomic signature, and upregulates key regulatory proteins of the glucose and lipid metabolism, immune system, circadian clock, DNA repair, cytoskeleton remodeling and cognitive function in healthy people^26^. Further, distinct metabolomic changes have been reported upon the observance of RIF^27^. Such proteomic and metabolomic changes reported upon RIF are inevitably reflected in the lipidomic mapping in the human body, which requires further investigation using advanced lipidomics techniques. To understand the nature of metabolic changes accompanying fasting and feeding, it is of paramount significance to examine the lipidomic changes in these physiological states.

Considering the extended fasting duration, the accompanying metabolic switches presented in the shift from glucogenic to ketogenic pathway^28^, and changes in circadian rhythm hormones^29^; IF is expected to be entailed with a list of lipidomic changes^30^. According to the available literature and the best of our knowledge, none of the published work had examined the lipidomics changes associated with the practice of RIF. Therefore, the current study was designed to investigate the lipidomic changes, expressed in terms of ceramides, sphingolipids, inflammatory markers, and lipid profiles associated with the practice of RIF in metabolically healthy people with overweight and obesity.

## Subjects, materials, and methods

### Study design

**Figure 1:**
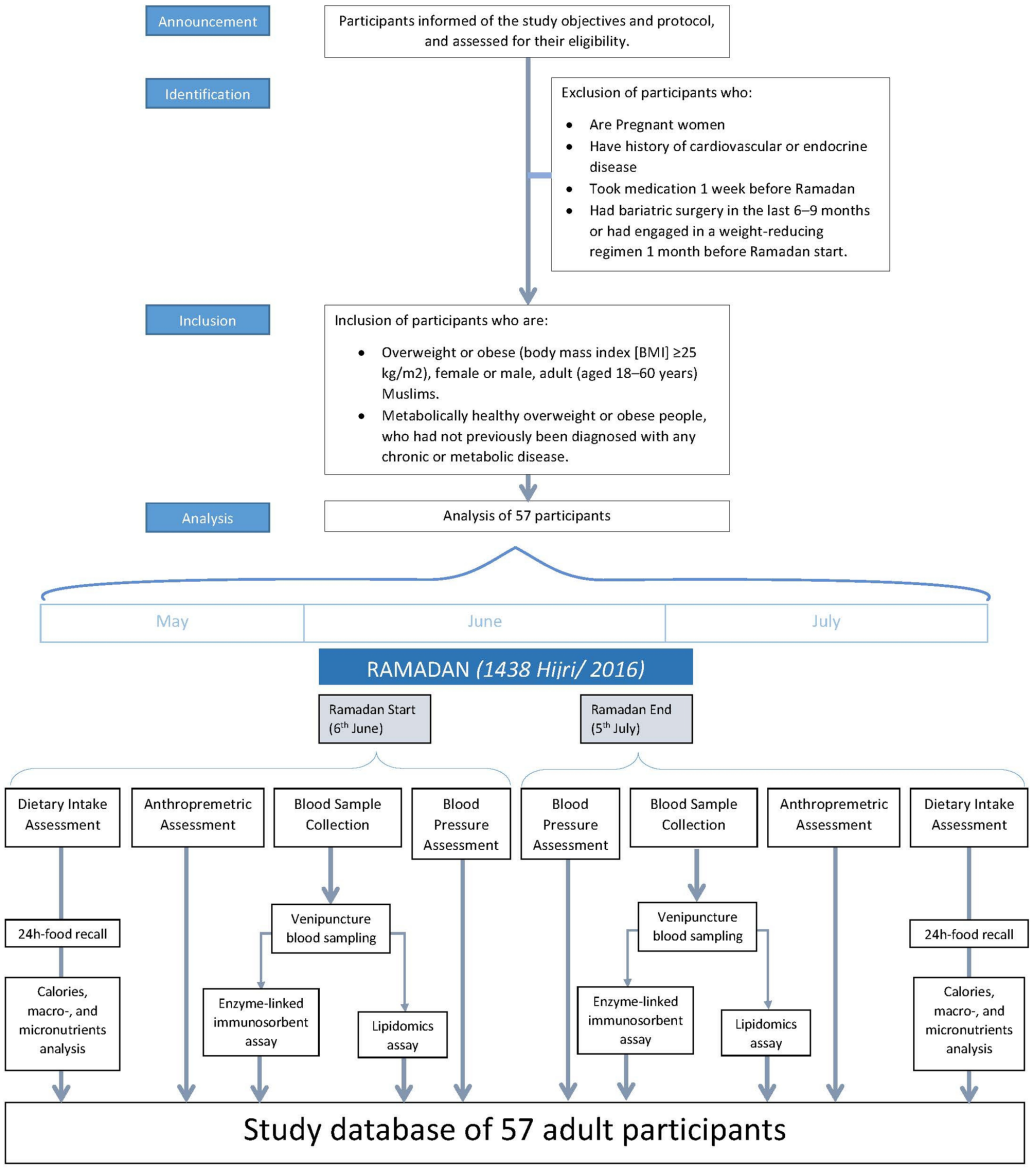
Flowchart of participants’ recruitment and outcome analysis and assessment steps.

This is a prospective cohort study executed during Ramadan month of the Arabic *Hijri* lunar calendar of 1438 (from June 2016 to July 2016). Data were collected 1 week before Ramadan (baseline or pre-fasting) and at the end of Ramadan (after completing 29–30 days of the fasting month). During Ramadan, fasting people refrain from drinking, including water, and food, and do not smoke or engage in sexual activity from dawn to sunset. The daily fasting duration in this study was approximately 15 h. We compared the studied variables for each participant before and after or at the end of Ramadan, meaning each participant served as his control. Participants did not receive any recommendations for dietary, lifestyle, or physical activity changes at any stage during this study. The jurisprudence rules of Ramadan excuse females from commencing fasting during their menstruation; therefore, the fasting days for female participants were about 23–25 days. Since physical activity may interfere with the subjects’ biochemical measurement and body composition by the end of RIF, study subjects were instructed to keep on their habitual physical exercise levels before and during Ramadan. A flowchart of the recruitment and analysis steps is shown in Fig. 1.

### Subjects

This observational study included a convenience sample of adults from the United Arab Emirates (UAE), including expatriates from Leavant countries (Palestine, Syria, Jordan, and Lebanon), Sudan, and Egypt who were residing in Sharjah. Potential participants were invited to enroll in this study through personal communications, hospital bulletin, and social media. The announcement included the inclusion criteria (male/female, healthy without predetermined chronic disease, or enrolled in a weight-reducing regime 1 month before the commencement of Ramadan). Those who were interested in the study contacted us and were asked to have a formal meeting at Sharjah University Hospital. This meeting involved informing participants about the study objectives and protocol, examining participants’ medical status and eligibility, letting them sign the informed consent, and taking the pre-fasting baseline measurements. Participants had 100% compliance with the study requirements and all of them had completed the study in the two phases of the study. Inclusion criteria were healthy female or male adults (aged 18–60 years) who had not previously been diagnosed with any metabolic or chronic disease. A set of inclusion and exclusion criteria were applied in recruiting the study participants (Fig. 1). The University of Sharjah Research and Ethics Committee (REC-16-05-11-01) approved this study, and this study abided by the Declaration of Helsinki principles. All participants signed a consent form before being enrolled in this study.

### Anthropometric assessment

Body weight (to the nearest 0.1 kg), height (to the nearest 0.1 cm), and waist circumference (WC) (to the nearest 0.1 cm), were measured using standardized protocols^31^, such as WHO 2008 reported guidelines. Body mass, without excess clothes, was measured using a body weight scale (Detecto, MO/USA), while stature was measured by a portable stadiometer (Seca GmbH and Co., Germany Model 217) without shoes. WC was measured at the umbilicus with participants in a standing position and breathing normally. BMI (kg/m^2^) was calculated accordingly. Diastolic blood pressure (DBP) and SBP were measured with participants in a sitting position using a digital upper arm monitor (Omron Healthcare Inc., Japan Model BP742N).

### Biochemical assessment

A venous blood sample (10 ml) was drawn from all participants 8–10 h after starting the fast (approximately between 11 a.m. and 1 p.m.). Within three hours of blood collection, samples were centrifuged, separated, and frozen at – 80 °C. The plasma inflammatory markers tumor necrosis factor-alpha (TNF-α), interleukin-6 (IL-6), and IL-10 were quantified using enzyme-linked immunosorbent assay (ELISA) reagents (Elabscience, USA and Aviscera Bioscience Inc., CA, USA).

### Dietary intake assessment

Utilizing the 24 h food recall technique, dietary intake was assessed. Dietitians gathered dietary data for three days (two weekdays and one weekend day) before and after or after Ramadan. Using two-dimensional food models, participants were able to recall and approximate the food portion sizes they consumed. Using Food Processor software (version 10.6 ESHA Research, Salem, OR, USA), total caloric, macro-, and micronutrient intakes were determined.

### Lipidomics assay

Human plasma samples were used for lipid extractions as previously described^5^. Briefly, The internal standard (IS) stock solution containing 17:0-sphingomyelin (d18:1/17:0) (2502 pmol/sample), dihydro-cer (d18:0/18:1) (5 pmol/sample), 16:0-ceramide (d18:1-d7/16:0) (6 pmol/sample), 18:0-ceramide (d18:1-d7/18:0) (2 pmol/sample), 24:0-ceramide (d18:1/24:0) (152 pmol/sample), 24:1-ceramide (d18:1/24:1) (20 pmol/sample), and 17:0-glucosylceramide (d18:1/17:0) (50 pmol/sample) was prepared in methanol. Serum samples were thawed on ice and vortexed before the lipid extraction. Samples were extracted in individual tubes, including a process blank. Serum (50 µL) was diluted in 133 µL of PBS and mixed with 225 µL of MeOH containing internal standards. The PBS-Serum-IS solution was then vortexed and 750 µL of methyl-tert-butyl-ether was added. We then incubated the sample on ice for 15 min, vortexing every 5 min. The solution was then centrifuged at 21,000 rcf and the top lipid-containing MTBE layer was transferred to a new tube. We then added an additional 750 µL of MTBE, incubated for 15 min on ice, vortexing every 5 min, and transferred the lipid-containing MTBE to the new tube. The lipid-containing MTBE was then dried down using a SpeedVac. The sample was then resuspended in 150 µL of HPLC grade isopropyl alcohol (IPA): acetonitrile (ACN): dd-H_2_O (8:2:2). We then sonicated and vortexed the tube to resuspend the solution. The samples were stored at 4°C preceding liquid chromatography-tandem mass spectrometry (LC-MS/MS) analysis.

Lipid extracts were separated on an Acquity UPLC CSH C18 1.7 μm 2.1 × 50 mm column, maintained at 60 °C. It was connected to an Agilent HiP 1290 Sampler and Agilent 1290 Infinity pump and equipped with an Agilent 1290 Flex Cube and Agilent 6490 triple quadrupole (QqQ) mass spectrometer. Quantification of sphingolipids was based on peak area ratios to the standards applied to the extracts. Lipid extracts are separated on an Acquity UPLC CSH C18 1.7 µm 2.1 × 50 mm column maintained at 60 °C connected to an Agilent HiP 1290 Sampler, Agilent 1290 Infinity pump, equipped with an Agilent 1290 Flex Cube and Agilent 6490 triple quadrupole (QqQ) mass spectrometer. Sphingolipids are detected using dynamic multiple reaction monitoring (dMRM) in positive ion mode. Source gas temperature is set to 210 °C, with a gas (N2) flow of 11 L/min and a nebulizer pressure of 30 psi. The sheath gas temperature is 400 °C, sheath gas (N2) flow of 12 L/min, capillary voltage is 4000 V, nozzle voltage is 500 V, high-pressure RF 190 V and low-pressure RF is 120 V. Injection volume is 2 µL and the samples are analyzed in a randomized order with the pooled QC sample injection eight times throughout the sample queue. Mobile phase A consists of ACN: H2O (60:40 v/v) in 10 mM ammonium formate and 0.1% formic acid, and mobile phase B consists of IPA: ACN: H2O (90:9:1 v/v) in 10 mM ammonium formate and 0.1% formic acid. The 5 chromatography gradient starts at 15% mobile phase B, increases to 30% B over 1 min, increases to 60% B from 1 to 2 min, increases to 80% B from 2 to 10 min, and increases to 99% B from 10 to 10.2 min where it’s held until 14 min. Post-time is 5 min and the flow rate is 0.35 mL/min throughout. Collision energies and cell accelerator voltages were optimized using sphingolipid standards with dMRM transitions as [M + H] +  → [m/z = 284.3] for dihydroceramides, [M + H] +  → [m/z = 287.3] for isotope-labeled dihydroceramides, [M-H2O + H] +  → [m/z = 264.2] for ceramides, [M − H2O + H] +  → [m/z = 267.2] for isotope-labeled ceramides and [M + H] +  → [M − H2O + H] + for all targets. Sphingolipids without available standards are identified based on HR-LC/MS, quasi-molecular ion, and characteristic product ions. Their retention times are either taken from HR-LC/MS data or inferred from the available sphingolipid standards. Results from LC-MS experiments are collected using the Agilent Mass Hunter Workstation and analyzed using the software package Agilent Mass Hunter Quant B.07.00. Sphingolipids are quantitated based on peak area ratios to the standards added to the extracts. Data processing was performed using Multiquanta™ Software for post-data acquisition and MarketView™ Software to perform statistical analysis.

## Statistical analysis

Statistical analyses were performed using SPSS software version 26 (IBM, Armonk, NY, USA). Continuous variables were described using mean ± standard deviation (SD), while categorical variables were described as the frequency of occurrence and percentage (for sociodemographic data. The paired sample t-test was used to compare pre-Ramadan and end-Ramadan anthropometric, biochemical, and dietary values. The correlation was used to study the effect of sphingolipid change on inflammatory biomarkers. The threshold for significance was *P* < 0.05. Our Bioinformatics analysis was performed using SPSS version 17.0.

## Results

### Description of the study sample

Results of the current observational study were reported following the STROBE (Strengthening the Reporting of Observational Studies in Epidemiology) statement^32^.

A cohort of 57 adults (40 males, 70%, and 17 females, 30% of the sample size) participated in this observational study. Supplemental Table S1 summarizes the sociodemographic characteristics of the participants. The mean age was 38.42 ± 11.18 years, with an age range of 18–58 years. Mean body weight was 88.32 ± 16.24 kg, with 23 (40.4%) being obese and 34 (59.7%) being overweight body weight. Before Ramadan fasting the SBP and DBP for all participants were 123.66 ± 12.21 and 72.43 ± 8.99 mmHg respectively. Males had SBP of 124.87 ± 11.97 mmHg and DBP of 73.64 ± 8.44 mmHg, whereas females had SBP and DBP of 120.82 ± 12.65 mmHg and 69.59 ± 9.84 mmHg, respectively.

A comparison of some anthropometric measurements before Ramadan and at the end of Ramadan is shown in Supplementary Table S2. There was a significant reduction (*P* = 0.001) in body weight (86.73 ± 15.74 kg vs. 88.32 ± 16.24 kg), body mass index (BMI, kg/m^2^) (29.40 ± 4.94 vs. 29.89 ± 5.02), body fat percent (28.58 ± 7.34% vs. 29.51 ± 7.09%), fat mass (25.25 ± 9.40 kg vs. 26.51 ± 9.49 kg) at the end of RIF when compared to before Ramadan, respectively. In addition to that, a significant decrease (*P* < 0.05) was shown in WC and hip circumference (HC) (97.23 ± 13.03 cm, 108.55 ± 8.87 cm, respectively), in comparison with before Ramadan (98.64 ± 13.69 cm, 110.08 ± 9.46 cm, respectively).

### Plasma lipid profile

**Figure 2:**
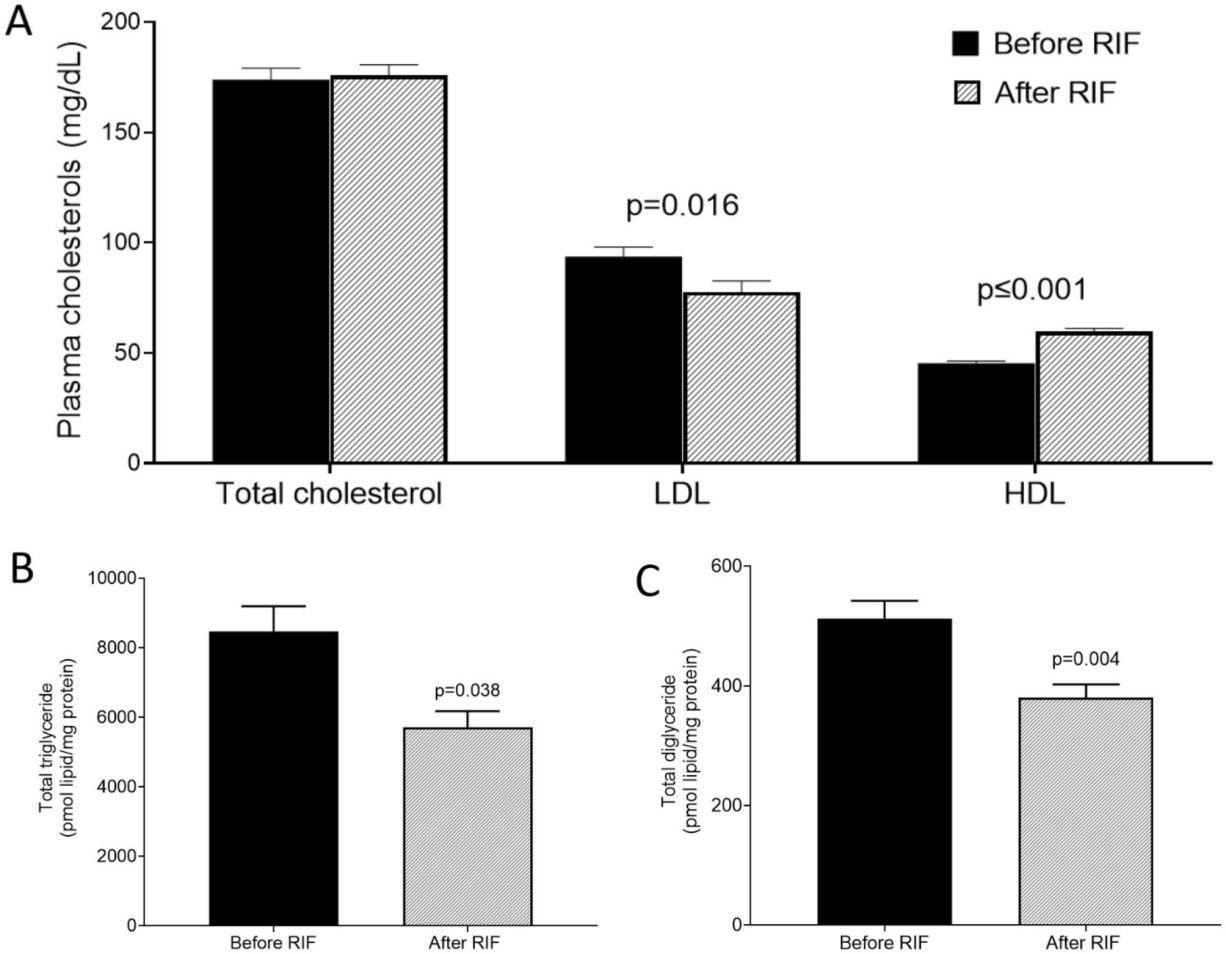
Impact of Ramadan intermittent fasting on plasma cholesterols, triglycerides, and diglycerides. (**A**) Plasma total cholesterol, low-density lipoprotein, and high-density lipoprotein, (**B**–**C**) plasma total triglycerides and diglycerides before and after Ramadan intermittent fasting were measured by mass spectrometry. Data are shown as mean ± SEM, n = 57.

To examine the impact of RIFon plasma lipids, we measured plasma TC, LDL-C, and HDL-C before and after RIF. Although RIF did not alter plasma TC, we found a significant decrease in LDL-C (*P* = 0.013) and an increase in HDL-C *(P* = 0.001) (Fig. 2A), suggesting an overall improvement in lipid profile. We next assessed plasma total TG and total DG from the dataset obtained from the lipidomics study. RIF was associated with significant reductions in both total TG (Fig. 2B; *P* = 0.038) and total DG (Fig. 2C; *P* = 0.004). Taken together, these results suggest that RIF was associated with improved plasma TC sub-types, TG, and DG.

### Inflammatory markers

**Figure 3:**
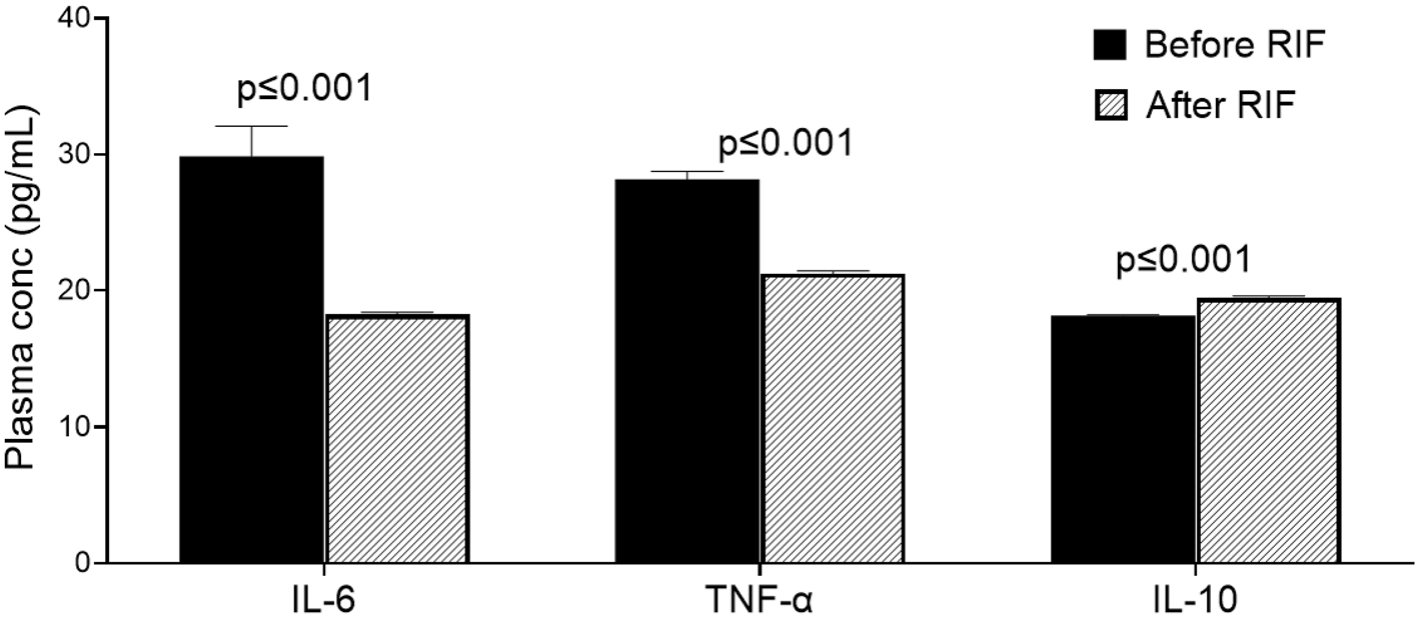
Effects of Ramadan intermittent fasting on markers of inflammation. Plasma interleukin-6, tumor necrosis factor-alpha, and interleukin-10 concentration before and after Ramadan intermittent fasting. Data are shown as mean ± SEM, n = 57.

We evaluated the impact of RIF on plasma inflammatory markers IL-6 and TNF-α. We found that the RIF was associated with significant reductions in both IL-6 and TNF-α (Fig. 3; both *P* = 0.001). Furthermore, we measured the plasma anti-inflammatory marker IL-10 and found that RIF was associated with a significant increase in plasma IL-10 levels (Fig. 3, *P* = 0.001). Together, these results display that RIF is associated with attenuated systemic low-grade inflammation.

### Dietary measurements

The changes in the intake of some macro- and micronutrients of the participants upon the observance of RIF are shown in Supplementary Table S3. There was a significant reduction (*P* = 0.002) in total protein intake at the end of Ramadan (89.93 ± 39.25 g/day) when compared to before Ramadan value (108.35 ± 36.61 g/day). However, there was a significant increase (*P* = 0.001) in total sugar intake at the end of Ramadan (107.73 ± 53.71 g/day) when compared to before Ramadan value (65.90 ± 31.33 g/day). A significant increase (*P* = 0.016) in intake was also marked in PUFA at the end of Ramadan (16.32 ± 16.81 g/day) when compared to before Ramadan value (10.47 ± 8.09 g/day).

A significant reduction (*P* = 0.001) was shown when comparing the dietary cholesterol intake at the end of Ramadan (272.56 ± 181.90 mg/day) when compared to before Ramadan (395.85 ± 176.89 mg/day). A significant increase (*P* = 0.006) was reported in vitamin C intake at the end of Ramadan (97.43 ± 66.71 mg/day) when compared to before Ramadan (73.50 ± 50.63 mg/day). Also, a significant increase was found when comparing the value obtained at the end of Ramadan with the before Ramadan value in omega 3 (*P* = 0.001) (1.82 ± 2.29 g/day and 0.66 g/day ± 0.56, respectively), lycopene (*P* = 0.048) (6769.76 ± 11,307.11 µg/day and 1798.96 ± 3382.99 µg/day, respectively) and in vitamin E intake (*P* = 0.042) (8.49 ± 9.44 mg/day and 5.75 mg/day ± 3.57, respectively). On the other hand, there was no significant change upon RIF in the intake of total calories, fat calories, total carbohydrate, total fat, saturated fat, water, MUFA, trans fat, alpha-carotene, β-carotene, omega-6, or selenium.

### Sphinganine and sphingosine

**Figure 4:**
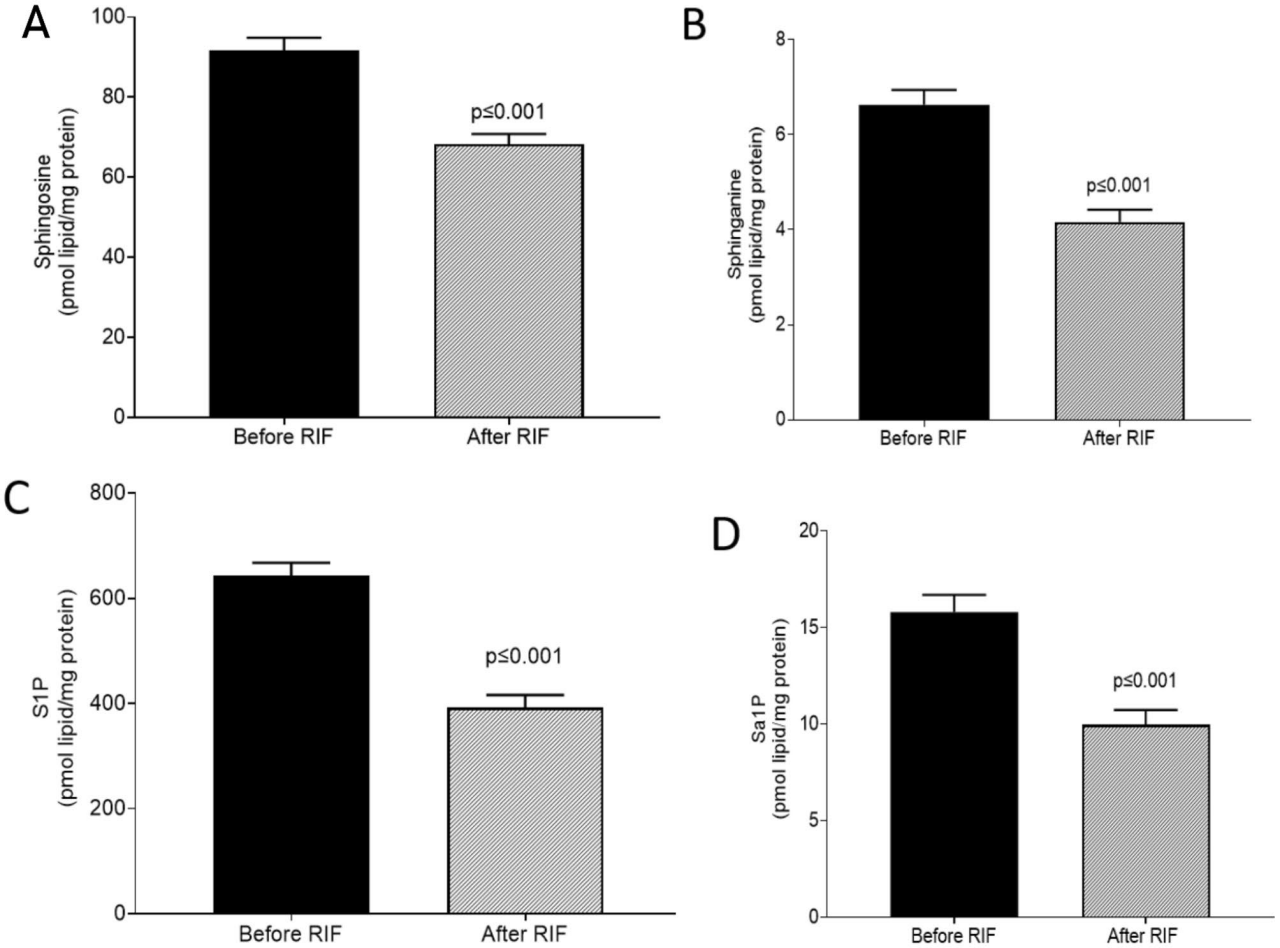
Impact of Ramadan intermittent fasting on sphingosine, sphinganine, S1P, and Sa1P. (**A**) Plasma sphingosine (**B**) plasma sphinganine, (**C**) plasma sphingosine-1-phosphate, and, (**D**) plasma sphinganine-1-phosphate before and after Ramadan intermittent fasting. Data are shown as mean ± SEM, n = 57.

Taking advantage of our large lipidomics dataset, we next assessed the changes in plasma sphingosine and sphinganine upon the observance of RIF. Our results revealed that RIF was associated with significant reductions in both plasma sphingosine (Fig. 4A; *P* ≤ 0.001) and sphinganine (Fig. 4B; *P* ≤ 0.001). Further, RIF was found to be associated with significant reductions in both plasma sphingosine-1-phosphate (S1P) and sphinganine-1-phosphate (Sa1P) (Fig. 4C,D; *P* ≤ 0.001). Collectively, these results demonstrated that the practice of RIF was associated with decreased levels of plasma sphingosine and sphinganine lipid species.

### Plasma sphingomyelin and ceramide species

**Figure 5:**
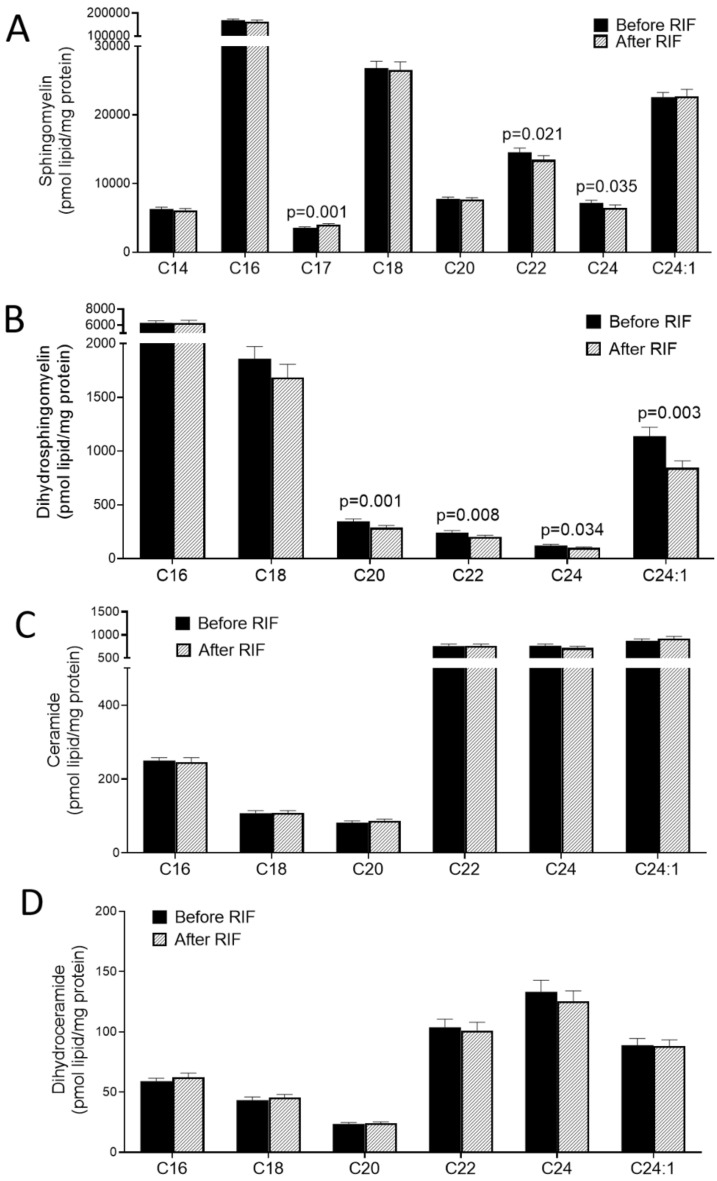
Impact of Ramadan intermittent fasting on sphingomyelin and ceramide species. (**A**) Plasma sphingomyelin species, (**B**) plasma dihydrosphingomyelin species, (**C**) plasma ceramide species, and, (**D**) plasma dihydroceramide species before and after Ramadan intermittent fasting. Data are shown as mean ± SEM, n = 57.

**Figure 6:**
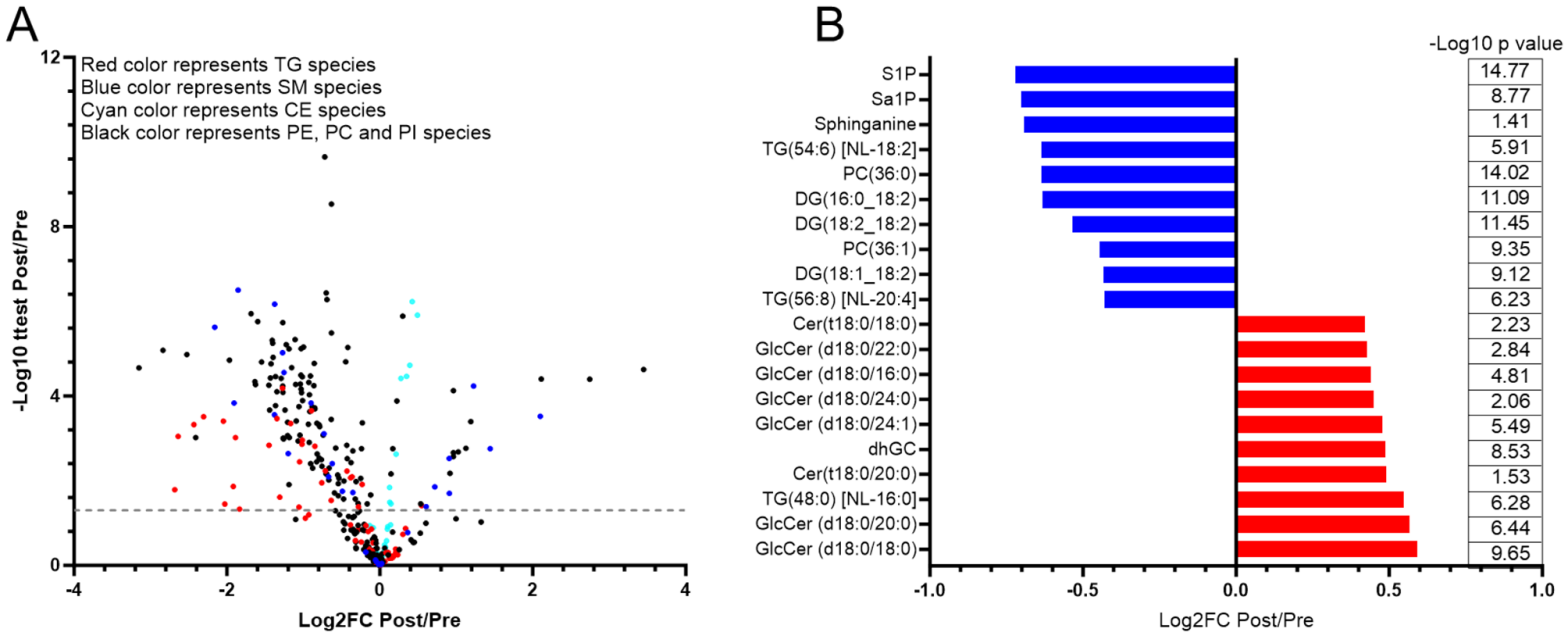
Global changes in plasma lipid species induced by Ramadan intermittent fasting. (**A**) Log2 fold changes of plasma lipid. (**B**) Top ten downregulated (blue) and top ten upregulated (red) lipid species. Group differences were assessed using paired *t*-test. n = 57.

We also examined the changes associated with the practice of RIF on plasma sphingomyelin (SM) and ceramide species. RIF was found to be associated with reduced C17, C22, and C24 SM (*P* = 0.001, 0.021, and 0.035 respectively), but not C14, C16, C18, C20, C24:1 SM (Fig. 5A). Likewise, RIF was associated with reduced C20, C22, C24, and C24:1 dihydrosphingomyelin (DhSM) (*P* = 0.001, 0.008, 0.035, and 0.003 respectively), but not C16 and C18 DhSM (Fig. 5B). However, in this study, we did not find any significant changes associated with the observance of RIF in plasma ceramides and dihydroceramides (DhCer) (*P* ≥ 0.11) (Fig. 5C,D). Taken together, these results demonstrated that RIF was associated with decreased levels of plasma SM and DhSM but not ceramides and DhCer. We have also analyzed the data in an unbiased manner and found robust global changes in plasma lipids due to RIF (Fig. 6A,B).

The correlation between ceramides sphingolipids as independent variables and inflammatory markers as dependent variables, before and at the end of Ramadan, is shown in Supplementary Table S4. A strong positive and significant correlation was found between DhCer and IL-10 before Ramadan (+ 0.538, *P* = 0.001). There was also a positive significant correlation between dihydroglucosylceramide (DhGC) and IL-10 before Ramadan (+ 0.329, *P* = 0.031), while there was a positive significant correlation between phytoceramides (Phyto) and IL-10 at the end of Ramadan (+ 0.296, *P* = 0.057). The DG was positively correlated with TNF-α before Ramadan (+ 0.539, *P* = 0.01), IL-10 at the end of Ramadan (+ 0.347, *P* = 0.024), and with TNF-α at the end of Ramadan (+ 0.396, *P* = 0.009). The TG is correlated positively with IL-10 before Ramadan (+ 0.361, *P* = 0.017) and at the end of Ramadan (+ 0.318, *P* = 0.040), and the TG also correlated positively with TNF-α before and at the end of Ramadan (+ 0.368, *P* = 0.015 and + 0.461, *P* = 0.002, respectively). The was no correlation between Cer, glucosylceramide (GC), DhSM, SM, and PC with any of the inflammatory markers both before and at the end of Ramadan. Supplementary Table S5 shows a heatmap of the correlation between ceramides sphingolipids and inflammatory markers (IL-10, IL-6, and TNF-α) at the end of Ramadan, with adverse correlations found mainly in IL-6. The bold correlations were significant, and the strongest correlation was found between TG and TNF-α inflammatory marker.

## Discussion

This study aimed to examine the extent to which the observance of RIF is associated with changes in plasma lipid profile, ceramides, sphingolipids, and inflammatory markers in overweight and obese subjects. The major novel findings of this study are as follows: (1) RIF is associated with reduced body mass and fat mass independent of changes in calorie intake, (2) this fasting regimen is associated with attenuated plasma TC, TG, and DG, (3) RIF is associated with reduces plasma sphingosine, sphinganine, S1P, and Sa1P, (4) RIF is associated with reduced plasma SM and DhSM, but not ceramide and DhCer species and (5) RIF is associated with attenuated inflammatory markers. To our knowledge, this is the first study demonstrating that practicing RIF is associated with reduced specific plasma sphingolipids, lipid profile, and systemic inflammation in overweight/obese subjects using lipidomic analysis. However, our observations do not reflect potential alterations in lipid flux. A better understanding of flux in these pathways will require future studies. Findings from this study support that the model of diurnal IF could be a strategy to combat the epidemic of overweight and obesity-associated impairments in lipid metabolism and systemic inflammation and the resultant cardiometabolic multimorbidity^33^.

Dyslipidemia is one of the major risk factors for the development of cardiometabolic diseases such as diabetes and atherosclerosis^34,35^. Our findings demonstrated that RIF was associated with significantly reduced LDL-C, TG, and DG levels while associated with a significant increase in HDL-C (*P* = 0.001) levels after 30 days of fasting in overweight and obese individuals. This indicates that fasting may contribute to the prevention of dyslipidemia and trigger weight loss by attenuating the lipid profile at the lipidomic level. These results are in agreement with the findings of a recent study that demonstrated that fasting during Ramadan was associated with improved cardiometabolic risk factors by significantly reducing serum TC (*P* < 0·0001) and TG (*P* = 0·0003) levels while significantly increasing HDL-C (*P* = 0.0001) after 25 days of fasting^36^. Furthermore, our findings are also supported by a previous study, which reported that TC, LDL-C, TG, and apoprotein A were significantly improved after Ramadan fasting in 84 patients with stable ischemic heart disease^37^. Papazoglou et al. also demonstrated that Ramadan fasting imposed significant reductions in LDL-C and TG levels in 30 patients with dyslipidemia^38^.

Abnormal regulation of fat metabolism may induce an inflammatory response during diet-induced obesity. Obesity-associated inflammation predisposes to the pathogenesis of atherosclerosis which triggers vascular inflammation in metabolic diseases^39^. IL-6 and TNF-α are the most attributable pro-inflammatory cytokines that enhance atherosclerosis and metabolic dysregulation in obesity^40^. On the other hand, IL-10 is an anti-inflammatory cytokine used by immune cells. However, IL-10 derived from immune cells can suppress thermogenesis and energy expenditure in adipocytes that drive insulin resistance in obesity^41^. Herein we show that RIF is associated with significantly reduced pro-inflammatory cytokines (IL-6 and TNF-α), while IL-10 was increased significantly. Interestingly, the reduction in TNF-α is supported by a previous study, in which it was demonstrated that TNF-α was reduced after Ramadan fasting both at serum and mRNA levels^36^. Moreover, other studies conducted in Germany and the United Arab Emirates (UAE) showed that IL-6 and TNF-α were significantly reduced at the end of Ramadan, while IL-10 was increased but did not reach a significant level in the German study^42^, but it was significantly increased in the UAE study^43^.

Systemic low-grade inflammation is a common underlying factor in the development of obesity and the subsequent affluent cardiometabolic diseases that are now prevalent, including cardiovascular disease, type 2 diabetes, and non-alcoholic fatty liver disease^44^. Sphingolipid metabolism has a vital role in the regulation of inflammatory signaling pathways in the human body by working as the second messenger that propagates the inflammatory response^45^. The current study showed significant reductions in the lipid profile components including LDL-C, total TG, total DG, sphingosine, sphinganine, S1P, Sa1P, SM (C17, C22, C24), and DhSMs (C20, C22, C24, C24:1). These improvements in the lipid profile components and sphingolipids at the end of the observance of RIF was concomitant with a significant reduction in the proinflammatory cytokines (IL-6 and TNF-α) and a significant increase in the anti-inflammatory cytokine IL-10. The observed reduction in these metabolic factors is consistent with the evident reductions in the cardiometabolic risk factors^22^, metabolic syndrome components^18^, and inflammatory and oxidative stress factors (TNF-α, IL-6, IL-1, *hs-*CRP, malondialdehyde or MDA) upon Ramadan fasting, as evident in recent systematic reviews and meta-analyses^18,19,22^.

Sphingosine can only be produced by the hydrolysis of ceramide, while sphingosine phosphorylation by sphingosine kinase produces S1P, which occurs at both the cytosol and plasma membrane. Ceramide can either be used for catabolism to generate sphingosine and sphingosine-1-phosphate or one of many complex sphingolipids with an additional head group^45^. In this present study, we demonstrate that IF during Ramadan is associated with attenuating sphingosine, sphinganine, sphingosine-1-phosphate, and sphinganine-1-phosphate. This treatment regimen also reduces the variety of SM (C17, C22, C24) and DhSM (C20, C22, C24, C24:1) species. These findings suggest that one or more acid ceramidase enzymes are down, a possibility that requires future investigations. Fasting is commonly associated with free fatty acid oversupply^46^ which may inhibit acid ceramidases^47^. The reduction in these lipid species may have implications for elevated skeletal muscle blood flow^48^ and contribute to improvements in metabolic function^28,47^, both of which require further elucidation in the RIF model.

It is noteworthy to remark that previous studies have revealed that the anthropometric and biochemical alterations resulting from Ramadan fasting can persist for up to one month following the conclusion of the fasting period^18^.

Lastly, the reported changes in dietary intake of sugars and other nutrients are consistent and repeatedly reported and mirror the reported changes in food groups and macronutrients by other research on Ramadan fasting^23,43,49^. The reported increased intake of simple sugars could be a response to the feeling of tiredness and inactivity at the end of the fasting hours. Further, eating simple sugars in the form of sweet palm dates is part of the prophetic guidance when Prophet Mohamed (Peace be Upon Him) encouraged fasting people to break their fasting with dates^50^.

Though the current work has several strengths such as being the first study with a sufficient sample size using high accuracy LC-MS machine on fresh blood samples, however, the current work entails several limitations that should be considered when discussing its results. The observational nature of the current work and the lack of experimentally controlled design make it difficult to infer causality. The use of the memory-based technique for assessing dietary intakes may impose some inaccuracies in dietary assessment results.

## Conclusions

Putting the previous findings together, the current study demonstrates that observance of RIF is associated with improvements in plasma sphingosine, sphinganine SM, and DhSM lipid species, along with improved lipid profile and inflammatory markers. It can be concluded, accordingly, that the model of RIF may impose short-term protection against cardiometabolic problems, and may be applied as a suggested cost-effective, health-improving, and cardiometabolic disease-preventing strategy for people with overweight and obesity.

### Supplementary Information

Supplementary Tables.

## Data Availability

The lipidomics data is attached and can be downloaded through the following repository link: https://docs.google.com/spreadsheets/d/1m7-NU26NTcOvqTl4MAbDE_rE-xxNoaQb/edit?usp=sharing&ouid=103274294772557410736&rtpof=true&sd=true. https://docs.google.com/spreadsheets/d/1iOeAibvfgvOIGvouxgJXfcQJ7DLn9_Ga/edit?usp=sharing&ouid=103274294772557410736&rtpof=true&sd=true.

## Abbreviations

IF: Intermittent fasting
RIF: Ramadan diurnal intermittent fasting
Cer: Ceramide
DG: Diglyceride
DhCer: Dihydroceramide
DhGC: Dihydroglucosylceramide
DhSM: Dihydrosphingomyelin
GC: Glucosylceramide
PC: Phosphatidylcholine
Phyto: Phytoceramides
SM: Sphingomyelin
TG: Triglyceride
TC: Total cholesterol
LDL-C: Low-density lipoprotein cholesterol
HDL-C: High-density lipoprotein cholesterol
