# Publisher Correction: Ramadan intermittent fasting is associated with ameliorated inflammatory markers and improved plasma sphingolipids/ceramides in subjects with obesity: lipidomics analysis

**DOI:** 10.1038/s41598-023-48849-0

**Published:** 2023-12-19

**Authors:** Mohamed Ibrahim Madkour, Md Torikul Islam, Trevor S. Tippetts, Kamrul H. Chowdhury, Lisa A. Lesniewski, Scott A. Summers, Falak Zeb, Dana N. Abdelrahim, Refat AlKurd, Husam M. Khraiwesh, Katia H. AbuShihab, Asma AlBakri, Khaled Obaideen, MoezAlIslam E. Faris

**Affiliations:** 1 Department of Medical Laboratory Sciences, College of Health Sciences, University of Sharjah, Sharjah, UAE; 2 Department of Nutrition and Integrative Physiology, University of Utah, Salt Lake City, UT USA; 3 Research Institute of Medical and Health Sciences (RIMHS), University of Sharjah, Sharjah, UAE; 4 Department of Nutrition, Faculty of Pharmacy and Medical Sciences, University of Petra, Amman, Jordan; 5 Department of Nutrition and Food Processing, Faculty of Agricultural Technology, Al-Balqa Applied University, Salt, Jordan; 6 Department of Nutrition and Food Technology, Faculty of Agriculture, The University of Jordan, Amman, Jordan; 7 Sustainable Energy and Power Systems Research Centre, RISE, University of Sharjah, Sharjah, UAE; 8 Department of Clinical Nutrition and Dietetics, College of Health Sciences, University of Sharjah, Sharjah, UAE

Correction to: *Scientific Reports* 10.1038/s41598-023-43862-9, published online 13 October 2023

In the original version of this Article MoezAlIslam E. Faris was incorrectly affiliated with ‘Department of Nutrition and Food Technology, School of Agriculture, The University of Jordan, Jordan, Amman’. The correct affiliations are listed below.

Research Institute of Medical and Health Sciences (RIMHS), University of Sharjah, Sharjah, UAE

Department of Clinical Nutrition and Dietetics, College of Health Sciences, University of Sharjah, Sharjah, UAE

In addition, the original version of this Article contained errors in Affiliations 4, 5, 6 and 8.

The correct affiliation 4 is:

Department of Nutrition, Faculty of Pharmacy and Medical Sciences, University of Petra, Amman, Jordan

The correct affiliation 5 is:

Department of Nutrition and Food Processing, Faculty of Agricultural Technology, Al-Balqa Applied University, Salt, Jordan

The correct affiliation 6 is:

Department of Nutrition and Food Technology, School of Agriculture, The University of Jordan, Jordan, Amman

The correct affiliation 8 is:

Department of Clinical Nutrition and Dietetics, College of Health Sciences, University of Sharjah, Sharjah, UAE

The original Article has been corrected.

