# Supplementary material for: Ramadan intermittent fasting is associated with ameliorated inflammatory markers and improved plasma sphingolipids/ceramides in subjects with obesity: lipidomics analysis
Source: Sci Rep. 2023 Oct 13;13:17322. doi: 10.1038/s41598-023-43862-9 (PMC10576029; doi:10.1038/s41598-023-43862-9)
Supplement: Supplementary file 1 — Supplementary Tables. [file 41598_2023_43862_MOESM1_ESM.docx]

**Supplementary Table S1.** Participants’ baseline sociodemographic, body weight, and blood pressure characteristics (n=57)

| **Characteristic** | | | | | | | **n (%)** | |
| --- | --- | --- | --- | --- | --- | --- | --- | --- |
| **Sex** | | | | Male | | | 40 (70.2%) | |
|  |  |  |  | Female | | | 17 (29.8%) | |
| **Body mass index (BMI, kg/m^2^)** | | | |  |  |  |  |  |
|  |  |  |  | Overweight (25 – 29.9) | | | 34 (59.7%) | |
|  |  |  |  | Obese (>30) | | | 23 (40.4%) | |
|  | | | | | | | | |
|  | | | **Minimum** | | **Maximum** | **Mean** | | **S.D.** |
| **Age (years)** | | | 18.00 | | 58.00 | 38.42 | | 11.18 |
| **Blood pressure (mmHg)*** | **Whole population** | SBP (mmHg) | 99.00 | | 157.00 | 123.66 | | 12.21 |
|  |  | DBP (mmHg) | 54.00 | | 87.00 | 72.43 | | 8.99 |
|  | **Male** | SBP (mmHg) | 100.00 | | 157.00 | 124.87 | | 11.97 |
|  |  | DBP (mmHg) | 54.00 | | 87.00 | 73.64 | | 8.44 |
|  | **Female** | SBP (mmHg) | 99.00 | | 138.00 | 120.82 | | 12.65 |
|  |  | DBP (mmHg) | 56.00 | | 87.00 | 69.59 | | 9.84 |

**Supplementary Table S2**. Changes in anthropometric variables before and at the end of Ramadan fasting month (n=57)

| **Parameter** | **Before Ramadan** | **End of Ramadan** | ***P-*value** |
| --- | --- | --- | --- |
| ***Anthropometric Variables*** | | | |
| Body weight (kg) | 88.32 ± 16.24 | 86.73 ± 15.74 | **0.001*** |
| Body mass index (kg/m^2^) | 29.89 ± 5.02 | 29.40 ± 4.94 | **0.001*** |
| Body fat percent (%) | 29.51 ± 7.09 | 28.58 ± 7.34 | **0.001*** |
| Fat mass (kg) | 26.51 ± 9.49 | 25.25 ± 9.40 | **0.001*** |
| Fat-free mass (kg) | 61.81 ± 10.37 | 60.94 ± 10.99 | 0.098 |
| Muscle mass (kg) | 58.73 ± 9.88 | 58.41 ± 9.57 | 0.092 |
| Visceral fat area (cm^2^) | 100.00 ± 48.59 | 96.77 ± 46.13 | 0.264 |
| Waist circumference (cm) | 98.64 ± 13.69 | 97.23 ± 13.03 | **0.030*** |
| Hip circumference (cm) | 110.08 ± 9.46 | 108.55 ± 8.87 | **0.001*** |
| Systolic blood pressure (mmHg) | 123.66 ± 12.21 | 125.64 ± 12.84 | 0.138 |
| Diastolic blood pressure (mmHg) | 72.43 ± 8.99 | 73.53 ± 10.26 | 0.329 |
| *Significant at P-value less than 0.001. | | | |

**Supplementary Table S3.** Changes in dietary variables before and at the end of Ramadan fasting month (n=57)

| **Parameter** | **Before Ramadan** | **End of Ramadan** | ***P-*value** |
| --- | --- | --- | --- |
| ***Dietary variables*** | | | |
| Total calories (kcal/day) | 2123.08 ± 754.20 | 2150.43 ± 847.11 | 0.829 |
| Fat calories (kcal/day) | 694.50 ± 396.38 | 687.07 ± 366.23 | 0.902 |
| Proteins (g/day) | 108.35 ± 36.61 | 89.93 ± 39.25 | **0.002*** |
| Total carbohydrates (g/day) | 253.66 ± 94.84 | 282.74 ± 122.88 | 0.080 |
| Total sugars (g/day) | 65.90 ± 31.33 | 107.73 ± 53.71 | **0.001*** |
| Total fats (g/day) | 77.32 ± 44.08 | 76.76 ± 41.10 | 0.934 |
| Saturated fats (g/day) | 23.49 ± 12.55 | 22.66 ± 12.20 | 0.667 |
| Total water intake (g/day) | 1397.02 ± 690.34 | 1518.62 ± 808.16 | 0.367 |
| MUFA (g/day) | 20.23 ± 11.24 | 23.05 ± 13.78 | 0.183 |
| PUFA (g/day) | 10.47 ± 8.09 | 16.32 ± 16.81 | **0.016*** |
| *Trans* fat (g/day) | 0.61 ± 0.82 | 0.65 ± 1.35 | 0.867 |
| Cholesterol (mg/day) | 395.85 ± 176.89 | 272.56 ± 181.90 | **0.001*** |
| Vitamin C (mg/day) | 73.50 ± 50.63 | 97.43 ± 66.71 | **0.006*** |
| Alpha carotene (µg /day) | 15.54 ± 26.85 | 22.84 ± 39.03 | 0.369 |
| Beta carotene (µg /day) | 393.88 ± 657.97 | 576.24 ± 938.61 | 0.209 |
| Omega 3 (~~m~~g/day) | 0.66 ± 0.56 | 1.82 ± 2.29 | **0.001*** |
| Omega 6 (~~m~~g/day) | 7.84 ± 7.08 | 10.06 ± 10.70 | 0.173 |
| Lycopene (µg/day) | 1798.96 ± 3382.99 | 6769.76 ± 11307.11 | **0.048*** |
| Selenium (µg/day) | 85.84 ± 48.50 | 70.98 ± 47.22 | 0.097 |
| Vitamin E (mg/day) | 5.75 ± 3.57 | 8.49 ± 9.44 | **0.042*** |
| *Significant at P-value less than 0.001. | | | |

**Supplementary Table S4.** Correlation between inflammatory markers (dependent factors) and sphingolipids (independent factors) before and at the end of Ramadan (n=57)

| **Ceramide/Sphingolipid** | **Before Ramadan**  ***r* (*P*-value)** | | | **End of Ramadan**  ***r* (*P*-value)** | | |
| --- | --- | --- | --- | --- | --- | --- |
|  | **IL-10** | **IL-6** | **TNF-α** | **IL-10** | **IL-6** | **TNF-α** |
| **Dihydroceramide**  **(DhCer)** | + 0.538  **(0.001) **** | + 0.081  (0.604) | -0.038  (0.808) | + 0.281  (0.071) | + 0.085  (0.591) | + 0.293  (0.060) |
| **Ceramide (Cer)** | + 0.012  (0.941) | + 0.112  (0.473) | + 0.186  (0.232) | + 0.131  (0.407) | -0.121  (0.445) | + 0.224  (0.154) |
| **Dihydroglucosylceramide (DhGC)** | + 0.329  **(0.031) *** | + 0.020  (0.896) | + 0.016  (0.917) | + 0.142  (0.370) | + 0.246  (0.116) | + 0.164  (0.298) |
| **Glucosylceramide**  **(GC)** | + 0.041  (0.794) | + 0.064  (0.682) | + 0.271  (0.079) | + 0.203  (0.198) | -0.006  (0.972) | + 0.226  (0.151) |
| **Dihydrosphingomyelin (DhSM)** | + 0.250  (0.106) | + 0.099  (0.526) | -0.095  (0.543) | + 0.212  (0.179) | +0.096  (0.545) | + 0.220  (0.161) |
| **Sphingomyelin (SM)** | -0.047  (0.764) | + 0.044  (0.777) | -0.043  (0.783) | + 0.142  (0.369) | -0.057  (0.721) | + 0.114  (0.474) |
| **Phytoceramide (Phyto)** | + 0.268  (0.082) | + 0.096  (0.539) | + 0.014  (0.928) | + 0.296  (0.057) | -0.042  (0.792) | + 0.082  (0.607) |
| **Diacylglycerol**  **(DG)** | + 0.111  (0.479) | + 0.240  (0.122) | + 0.539   - 1. ***** | + 0.347  **(0.024) *** | + 0.110  (0.487) | + 0.396  **(0.009) **** |
| **Triacylglyceride**  **(TG)** | + 0.361  **(0.017) *** | + 0.120  (0.443) | + 0.368  **(0.015) *** | + 0.318  **(0.040) *** | + 0.087  (0.583) | + 0.461  (**0.002) **** |
| **Phosphatidylcholine**  **(PC)** | 0.157  (0.314) | - 0.017  (0.912) | + 0.106  (0.497) | + 0.271  (0.082) | -0.030  (0.851) | + 0.157  (0.321) |
| *Significant at P-value less than 0.05. **Significant at P-value less than 0.001. | | | | | | |

**Supplementary Table S5.** Heatmap of the correlation between inflammatory markers (dependent factors) and ceramides/sphingolipids (independent factors) at the end of Ramadan (n=57)

| **Ceramide/Sphingolipid** | **IL-10** | **IL-6** | **TNF-α** |  |  |
| --- | --- | --- | --- | --- | --- |
| **Dihydroceramide (DhCer)** | 0.281 | 0.085 | 0.293 | Weakest Correlation |  |
| **Ceramide (Cer)** | 0.131 | -0.121 | 0.224 |  |  |
| **Dihydroglucosylceramide (DhGC)** | 0.142 | 0.246 | 0.164 |  |  |
| **Glucosylceramide (GC)** | 0.203 | -0.006 | 0.226 |  |  |
| **Dihydrosphingomyelin (DhSM)** | 0.212 | 0.096 | 0.22 | Strongest Correlation |  |
| **Sphingomyelin (SM)** | 0.142 | -0.057 | 0.114 |  | 6 |
| **Phytoceramide (Phyto)** | **0.296** | -0.042 | 0.082 |  | 7 |
| **Diacylglycerol (DG)** | **0.347** | 0.11 | **0.396** |  | 8 |
| **Triacylglyceride (TG)** | **0.318** | 0.087 | **0.461** |  | 9 |
| **Phosphatidylcholine (PC)** | 0.271 | -0.03 | 0.157 |  | 10 |
